# Hippocalcin: A New Solution to an Old Puzzle

**DOI:** 10.1016/j.neuron.2007.01.026

**Published:** 2007-02-15

**Authors:** David A. Brown, Barrie Lancaster, Mala M. Shah

**Affiliations:** 1 Department of Pharmacology, University College London, Gower Street, London, WC1E 6BT, United Kingdom

## Abstract

In hippocampal pyramidal neurons, calcium entry following an action potential burst results in a slow afterhyperpolarization (sAHP) that critically regulates subsequent excitability. Although this potassium current was described two decades ago, the mechanism whereby the rise in intracellular calcium generates the sAHP was, until now, not known. In this issue of *Neuron*, Tzingounis et al. now show that calcium binding to hippocalcin, a member of the NCS family, is one of the necessary steps involved in production of the sAHP.

## Main Text

In hippocampal and cortical pyramidal neurons, the calcium entry that accompanies a burst of action potentials triggers a long-lasting hyperpolarization called the slow afterhyperpolarization (sAHP) that lasts for several seconds. This is important physiologically because it inhibits subsequent action potentials and limits the frequency of repetitive action potential discharges (“spike frequency adaptation”). Furthermore, the sAHP is powerfully inhibited by several neurotransmitters, such as noradrenaline, acetylcholine, and serotonin, resulting in a strikingly enhanced neuronal excitability. This is thought to underlie some forms of learning and memory (Disterhoft et al., 2004). However, in spite of the fact that the sAHP, and the underlying potassium current (I_sAHP_), were first described two decades ago (Alger and Nicoll, 1980; Lancaster and Adams, 1986), the mechanism whereby calcium activates the current has remained a puzzle. The search for the solution is not helped by the fact that the molecular nature of the sAHP channels is not known (Stocker et al., 2004).

One of the most challenging features of I_sAHP_ is that, although the rise in intracellular calcium following an action potential is virtually instantaneous, the current itself requires several hundred milliseconds to reach its peak. Several suggestions as to why this should be so have been made, such as intermediation of a “second messenger” (Schwindt et al., 1988), buffered diffusion from the source of calcium to the responsive potassium channels (Lancaster and Zucker, 1994), delayed facilitation of the calcium current following an action potential burst (Marrion and Tavalin, 1998), and slow kinetic responses of the potassium channels to calcium (Sah and Clements, 1999).

The Nicoll lab, with their collaborators, have now returned to this question (Tzingounis et al., 2007), and have come up with a very interesting solution—namely, that there *is* a second messenger, in the form of the diffusible calcium binding protein hippocalcin. Their principal evidence for this is that I_sAHP_ was very substantially reduced in hippocampal neurons from mice in which the hippocalcin gene was disrupted. Though the authors do not measure Ca^2+^ transients directly, they do show that (in contrast to I_sAHP_), the current carried by another set of calcium-activated potassium channels, the SK2 (KCa2.2) channels (Stocker et al., 2004), was not reduced but actually enhanced in these knockouts. SK channels are activated directly by calcium through attached calmodulin molecules (Xia et al., 1998), and hence, unlike the sAHP channels, track the Ca^2+^ transient with reasonable fidelity. Thus, for SK channels, hippocalcin acts simply as an endogenous diffusible buffer, reducing submembrane Ca^2+^ by transporting it into the bulk cytoplasm—rather like BAPTA or EGTA, as the authors show.

**Figure 1:**
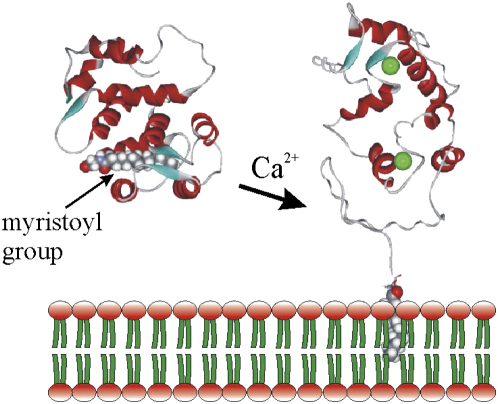
Structure of Myristoylated Recoverin In the resting state (left side) the N-terminal myristoyl group (arrow) is marked. Two Ca^2+^ ions (right side, green balls) bind to the EF-2 and EF-3 hands and induce a conformational change exposing the myristoyl group, which then inserts into the membrane. Reprinted from Burgoyne et al. (2004), with permission from Elsevier.

So what is hippocalcin and how does it work? Hippocalcin is a member of the neuronal calcium sensor (NCS) family of calcium binding proteins (Burgoyne et al., 2004), with four potential Ca^2+^ binding EF hands (though only two may be functionally occupied). The archetype of this family is the photoreceptor protein recoverin (also known as S-modulin or visinin), which mediates calcium-dependent inhibition of rhodopsin kinase and hence assists in the adaptation of the phototransduction pathway. This protein is myristoylated at the N-terminal, but the myristoyl group is normally buried within a hyrdophobic pocket (Figure 1, left side). However, when it binds Ca^2+^ (at EF hands 2 and 3), the myristoyl group “flips out”—a process termed a “myristoyl switch” (Ames et al., 1997); this promotes translocation to the cell membrane where the myristoyl moiety becomes embedded in the membrane lipid layer (Figure 1, right side).

Hippocalcin behaves similarly and rapidly translocates to membrane sites following a rise in intracellular Ca^2+^ (O'Callaghan et al., 2003). Indeed, such a translocation has been observed in hippocampal neurons during electrical activity (P. Belan et al., 2005, Soc. Neurosci., abstract), as Tzingounis et al. note. Furthermore, hippocalcin is strongly expressed in the hippocampus and cortex, and responds to Ca^2+^ within a range (200–800 nM; O'Callaghan et al., 2003) that matches the sensitivity of the AHP current.

Thus, the scenario would be that calcium enters the cytoplasm where it binds to hippocalcin, then induces a myristoyl switch whereupon calcium-bound hippocalcin translocates to (and inserts in) the membrane to generate the sAHP. In support of this myristoylation requirement, Tzingounis et al. go on to show that transfection of hippocalcin into cultured neurons generates an I_sAHP_, whereas a mutated form that could not be myristoylated did not.

Of course, as with any such novel hypothesis, there are some outstanding questions. For example, I_sAHP_ was not completely lost in the hippocalcin knockouts, even though (presumably) there was no hippocalcin (Kobayashi et al., 2005). Also, the residual current appeared strikingly insensitive to noradrenaline compared with that from wild-type mice. Assuming that the residual current is carried by the same channels, does this mean that there is a “reserve” second messenger (perhaps operating at higher levels of Ca^2+^, as Figure 3 in Tzingounis et al. might suggest)? And, if so, does this messenger act differently from hippocalcin, such as to occlude the effect (PKA-mediated phosphorylation) of noradrenaline? Or perhaps endogenous hippocalcin actually enhances the sensitivity to noradrenaline by inhibiting phosphodiesterase. [Haynes et al. (2006) have reported that hippocalcin binds to phosphodiesterase.] In this context, it would have been useful to know whether the residual current was equally insensitive to a transmitter acting through a different pathway, such as acetylcholine.

Finally, when hippocalcin gets to the membrane, how does it activate the sAHP channels? Does it interact directly with the channel? Or is a third messenger system involved? Hippocalcin binds strongly to phosphatidylinositol-4,5-bisphosphate (PIP_2_) (O'Callaghan et al., 2005), so one possibility is that the channels are held shut by membrane PIP_2_ and then open when hippocalcin sequesters channel-associated PIP_2_ molecules. The final resolution of these questions may have to wait until the molecular identity of the true sAHP channel is revealed. The availability of new and selective sAHP blockers (Shah et al., 2006) may help here. This new information about hippocalcin will be particularly crucial since we will no longer have to look for calcium-activated channels, but instead for channels that can be activated by hippocalcin.
